# Ultrasound Treatment in Berry Puree Production: Effects on Sensory, Rheological, and Chemical Properties

**DOI:** 10.3390/molecules31020260

**Published:** 2026-01-12

**Authors:** Jan Piecko, Monika Mieszczakowska-Frąc, Niall J. Dickinson, Anna Wrzodak, Karolina Celejewska, Michael Bom Frøst, Belinda Lange, Charlotte Dandanell, Jacek Lewandowicz, Patrycja Jankowska

**Affiliations:** 1 Fruit and Vegetable Storage and Processing Department, The National Institute of Horticultural Research, Konstytucji 3 Maja 1/3, 96-100 Skierniewice, Poland; monika.mieszczakowska@inhort.pl (M.M.-F.); niall.dickinson@inhort.pl (N.J.D.); anna.wrzodak@inhort.pl (A.W.); karolina.celejewska@inhort.pl (K.C.); 2 Department of Food Science, Design and Consumer Behaviour, University of Copenhagen, Rolighedsvej 26, 1958 Frederiksberg, Denmark; mbf@food.ku.dk (M.B.F.); belinda@food.ku.dk (B.L.); chd@food.ku.dk (C.D.); 3 Department of Food Concentrates and Starch Products, Prof. Wacław Dąbrowski Institute of Agriculture and Food Biotechnology—State Research Institute, Starołęcka 40, 61-361 Poznań, Poland; jacek.lewandowicz@ibprs.pl (J.L.); patrycja.jankowska@ibprs.pl (P.J.)

**Keywords:** haskap berry, strawberry, ultrasound, puree production, anthocyanins, viscosity

## Abstract

Berries are a valuable source of health-promoting substances, including vitamins, microelements, and polyphenols. Optimising the extraction efficiency of these compounds during processing is crucial to minimise their loss into the waste stream. Ultrasound technology is recognised as a sustainable and promising tool for improving extraction; however, previous literature has not sufficiently addressed the optimal point of its application in fruit puree processing, and its impact on the sensory properties of the final product has only occasionally been explored. As one of the first reports, this study aimed to determine the optimal moment for ultrasound application within a puree production scheme. In the second stage of the experiment, four recipes based on strawberry and haskap berry were tested. The results demonstrated the potential for enhancing sensory quality of puree by using an ultrasound treatment. It was found that the ultrasound-treated purees showed significantly higher pectin levels and improved rheological properties, while the content of anthocyanins and L-ascorbic acid remained mainly unchanged. This indicates that the non-thermal nature of ultrasound treatment can induce positive changes from a sensory and rheological point of view without causing the degradation of health-promoting compounds, offering a viable strategy for improving berry puree quality.

## 1. Introduction

Berries such as strawberries (*Fragaria × ananassa*) and haskap berries (*Lonicera caerulea*) are a rich source of health-promoting compounds and have high processing value [1,2]. The health-promoting properties of berries are primarily due to polyphenolic compounds, including anthocyanins [3], which are pigments that colour the skin of these fruits, usually dark blue. These fruits are also a source of numerous vitamins, microelements, and dietary fibre [4]. Alongside juices, the production of berry purees is one of the key methods of processing berry fruits, and in many cases, it is the optimal method [5]. Fruit purees, especially those made from berries, are a valuable component in the creation of other products, such as puree juices, i.e., smoothies, a range of other products, and are also recently sold as ready-to-eat products. Due to the level of nutrient transfer from the raw material to the final product, purees deserve much more attention than juices, as they usually contain significantly more dietary fibre and minerals [6]. Due to their short shelf life, berries undergo various preservation and processing methods, the main commercial applications of which are freezing, juice and purée production, drying, fermentation, and extract production [7]. However, in general, food processing causes a partial loss of bioactive compounds [8], and attempts to optimise the process are still being researched. One direction in production optimisation may be the use of ultrasound, which is successfully used both in analytical methods in laboratories and in food industry production [9]. The use of ultrasound in food processing is a versatile and highly effective technique used in industry to produce, among other things, uniform mixtures [10]. The basic mechanism is based on acoustic cavitation: high-frequency sound waves generate alternating cycles of high and low pressure in the liquid, causing microbubbles to form, expand, and collapse rapidly [11]. This process creates intense local shear forces, shock waves, and micro-jets that grind particles, break down cell walls, and disperse or emulsify even highly viscous and difficult-to-process liquids [10]. Based on literature data, the modification of rheological properties, including viscosity, particle size distribution, transformation, and pectin content, are among the most important parameters subject to change as a result of ultrasound application [12]. In terms of product bioactivity, carotenoids, anthocyanins, and ascorbic acid are among the compounds whose content may change under the influence of ultrasound [13]. Ultrasound treatment can also increase process efficiency [14] and therefore deserves attention, both for its economic value and for the current trend towards reducing waste production.

Although the fundamental mechanisms of US are well established, its specific application and optimisation parameters for raw materials such as haskap berry and strawberry remain underexplored, particularly in the context of minimising production waste and maximising the extraction of bioactive compounds from berry skins generated as by-products during puree production. Therefore, this study investigates the impact of ultrasound treatment of fruit pulp on the quality parameters of the resulting purees, with the aim of enhancing extraction efficiency and promoting beneficial rheological properties. The experimental design comprised two stages: first, to identify the optimal production step for ultrasound application, and second, to evaluate the effect of the composition of ultrasound-treated fruit pulp on the quality of the final strawberry–haskap berry puree. The obtained purees were analysed for L-ascorbic acid, anthocyanins, and total pectin content, while sensory attributes and rheological properties were also assessed.

## 2. Results and Discussion

### 2.1. Stage I of Experiment—Processing Method Selection

**Figure 1:**
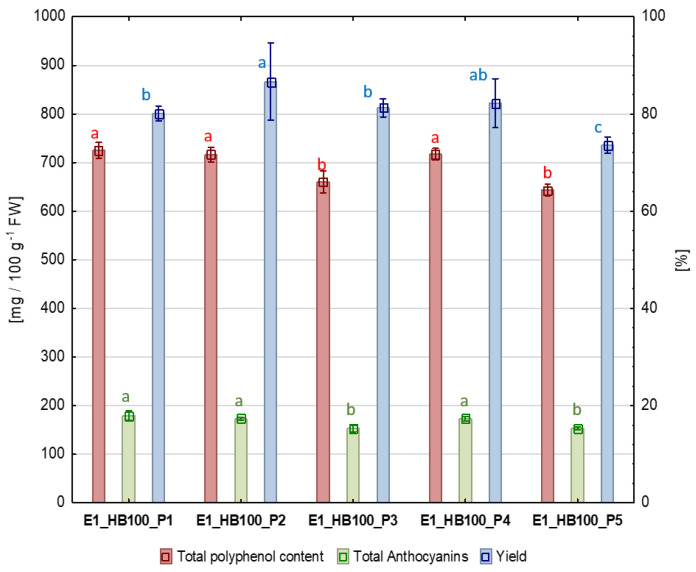
Processing yield [%], TPC (total polyphenols content), and TAC (total anthocyanins content) [mg 100 g^−1^ FW] in haskap berry purees using ultrasound. P1: puree without ultrasound treatment; P2: ultrasound treatment before pureeing; P3: ultrasound treatment before pureeing, without heating; P4: ultrasound treatment after pureeing; and P5: puree heated to match the temperature reached during ultrasound processing. Means marked with the same letter and in the same color do not differ significantly (*p* < 0.05) according to HSD Tukey’s test (*n* = 6).

Figure 1 below shows the results of the puree processing yield, total polyphenol content (TPC), and total anthocyanin content (TAC) in purees obtained from haskap berries using five production methods (P1–P5). The puree yield was calculated as the percentage of obtained puree weight compared to raw material weight. This value has the greatest economic significance because it directly reflects the efficiency of puree production. The P2 and P4 methods, in which US treatment was applied before or after pureeing, were characterised by the highest efficiency, amounting to 86.7 ± 2.6% and 82.2 ± 1.7%, respectively. Method P2 (US before pureeing) had an increase in efficiency of 6.6% compared to P1 (control). The puree yield obtained with method P4 did not differ significantly from the methods P1 (80.1 ± 0.5%) and P3 (81.2 ± 0.6%). A significantly lower yield was obtained with method P5 (77.4 ± 0.6%), which was not ultrasound-assisted and in which the pulp temperature was lower (50 °C). Replacing the heating stage (pulp heating up to 80 °C) with ultrasound treatment (method P3) did not result in higher efficiency compared to P1 (control). However, treatment P3 significantly increased the yield of the puree compared to the control sample P5. This observation clearly confirms that, in addition to the temperature effect, US definitely had a beneficial impact on processing yield. Comparing the highest- and the lowest-yielding methods (P2 and P5, respectively), the difference was 9.3%.

To determine the impact of ultrasound treatment on the content of health-promoting compounds, an analysis of TPC and TAC was performed (Figure 1). The TPC in objects P1, P2, and P4 did not differ significantly from each other and averaged 719.67 ± 13.74 mg 100 g^−1^ FW. The content of these compounds in this group was 17.8% higher than in objects P3 and P5. Methods P3 and P5 did not differ significantly from each other in terms of the content of these compounds and averaged 651.9 ± 18.38 mg 100 g^−1^ FW. Due to the higher susceptibility of anthocyanins to degradation under the influence of processing (mainly under the influence of temperature, oxygen, and light), they can be a good indicator of the overall loss of nutritional value of food. An analysis of TAC was performed to supplement the information on the quality of the purees obtained. Similar to TPC, objects P1, P2, and P4 did not differ significantly in terms of the amount of TAC, averaging 175.08 ± 6.40 mg/100 g^−1^ FW. The total anthocyanin content in objects P3 and P5 also did not differ significantly and averaged 153.10 ± 4.71 mg/100 g^−1^ FW. Neither adding US treatment to the production scheme nor replacing heating with ultrasound treatment resulted in higher TPC and TAC in the purees compared to control method (P1). Processing temperature had a greater positive impact on the content of these compounds than the use of ultrasound. There are some discrepancies here with regard to the available literature. In an article concerning the impact of ultrasound at different frequencies and power levels on enzyme activity and bioactive compounds in strawberry puree, the results indicated a high potential for this technology to replace temperature treatment [15], which was not reflected in the results we received. Furthermore, no negative impact of ultrasound was observed; in P4, where ultrasound was used as an additional treatment, there was no degradation of these compounds. A study examining the content of phenolic compounds at different stages of digestion of haskap berry puree showed that the use of ultrasound to prepare the puree increased the TPC and TAC of the digested samples, thereby enhancing their antioxidant capacity and antiproliferative activity [16]. An increase in TPC of digested samples was observed with increasing ultrasound power. In contrast, the opposite was observed for anthocyanins: the higher the power, the lower the TAC level. These findings, combined with the results obtained in our study, confirm the potential of ultrasound as a tool that can help combine economic and health-promoting aspects in berry processing. In studies on raspberry and blueberry purees, US had a slightly different effect depending on the raw material: it increased the total flavonoid content, reduced TAC, and increased TPC only in raspberry puree [17]. However, in this case, 400 watts of ultrasound power was applied to a 500 mL sample, and the purees were diluted 1:1 with distilled water before treatment. This reduced density may have increased the efficiency of US energy transfer to the sample, which may explain these observations. However, in a study using a completely different material—prebiotic soursop whey beverage—the total polyphenol content increased with increasing ultrasound power, despite a significant increase in temperature [18]. In this case, a power of 200 to 600 W was applied to a 25 mL sample, and the treatment lasted 3 min. Similarly, in experiments with fruit and vegetable juices (orange, lime, carrot, and spinach juice), ultrasound proved to be significantly better in terms of preserving TPC compared to thermal pasteurisation [19]. For a 250 mL sample, a power of 100 W was applied at a frequency of 20 kHz for 15 min, while maintaining the temperature at 30 °C. These experiments confirmed a similar observation that we noted in our work, namely that ultrasound itself does not have a negative effect on the TPC. This conclusion is true, but it has its limitations. In an experiment using a product slightly thicker than juice—a smoothie, and therefore somewhat more similar to the product tested in this study—US treatment for 1–10 min in some cases led to a reduction in TPC, especially at maximum amplitude values [20]. In this experiment, 9.2, 13.3, and 22.8 W cm^−2^ of ultrasound power were applied to a 200 mL of sample. Therefore, it can be assumed that changes in bioactive compounds depend largely on the parameters used in the experiments, particularly processing time and applied power. Clearly, the nature and properties of the matrix also have an impact on TPC levels following US treatment. The observed effect of ultrasound on the content of polyphenols and anthocyanins may also depend on the characteristics and content of other compounds in the treated material. The effect of AA content on anthocyanin degradation has been partially investigated [21], indicating that this process may be slowed down by its presence in the product. Therefore, AA content in the material may affect the observed anthocyanin degradation level. The effect of pH on anthocyanin extraction was also investigated. For example, in a study on anthocyanins extraction from jaboticaba berries, the optimal extraction conditions in an ultrasonic bath at a frequency of 40 kHz were a pH of 1.5 and a treatment time of 30 min [22].

Viscosity is one of the fundamental parameters determining the quality of fruit purees. Higher viscosity contributes to product stability during storage [23] and, as an important rheological property, also positively influences sensory perception [24]. Among the tested samples, P2 showed the highest apparent viscosity (1108 mPa·s), followed by P1 (698 mPa·s) (Table 1). The remaining purees (P3, P4, and P5) exhibited significantly lower values, ranging from 335 to 170 mPa·s. The obtained results suggest that the application of ultrasound as an additional processing step (method P2) may enhance the apparent viscosity of puree.

The available literature on this subject is limited. Much more often than purees, juices or nectars have been the subject of research concerning ultrasound treatment effects on viscosity. For example, in the case of mango nectar enriched with pectins, ultrasound treatment initially increases, but over time gradually decreases, the apparent viscosity [25]. Similar results were obtained for strawberry pulp [26], with sudden changes in rheological properties. In this case, the reduction in product viscosity was associated with a decrease in the molecular weight of pectin compounds. The observation was related to the degradation of water-soluble pectins. These results indicate that US treatment must be tailored to the desired effects. A decrease in the apparent viscosity of orange juice has also been observed with increasing ultrasound processing time [27]. Similar results have been reported for changes in the viscosity of tomato juice [28] and fruit smoothies, in cases which ultrasound treatment led to a reduction in product viscosity [20], especially at higher amplitudes and longer treatment times. The effect of ultrasound on the rheological properties and consistency of apple, cranberry, and berry juices has also been observed, and it can be concluded that ultrasound can shape rheological properties, but the effects depend on specific process settings like amplitude, duration, and treatment temperature [19].

By integrating the results of TPC, TAC, yield, and apparent viscosity of the obtained purees, the P2 method was selected as the most promising. Although it did not increase the content of bioactive compounds, it significantly improved the rheological properties of the puree sample and had a positive effect on yield. In the further part of the study, the results discussed will concern the comparison of the quality of the purees obtained by using this method (P2) with the control puree (obtained by the P1 method).

### 2.2. Result for Stage II of the Experiment—Evaluation of the Ultrasound Treatment for Different Recipes

#### 2.2.1. Microrheological Properties

Microrheological properties of purees were characterised by elasticity index (EI), fluidity index (FI), solid–liquid balance (SLB), and macroscopic viscosity index (MVI) (Table 2). The analysis was performed to compare the impact of US treatment and puree recipes on product properties that may affect, among other things, the sensory perception of puree and its stability. The above parameters refer to analyses performed on a microscopic scale and only partially and to a limited extent reflect the macroscopic properties of the products. The recipe and processing conditions caused significant changes in the viscoelastic behaviour of the samples studied. Strawberry puree (S100_P1) when compared to haskap berry puree (HB100_P1), had significantly higher EI and SLB (*p* < 0.05). If we also take into account the fact that this sample had a higher FI and lower MVI (although neither was statistically significant), we can conclude that parameters indicate liquid-like characteristics of the S100_P1 puree, although EI values still reflect relatively higher elasticity compared to the other samples. In the case of S60_HB40_P1 and S80_HB20_P1 purees, increasing the proportion of haskap berries led to a decrease in EI and an increase in MVI. This observation can be explained by the properties of the raw material: haskap berries have a significantly higher proportion of solid fragments, such as a much thicker skin than strawberry fruit. Thus, in the case of HB purees, suspended skin fragments and particle agglomerates may affect all microrheological results. The effect of US was also quite consistent for all samples regardless of their composition, leading to more liquid-like behaviour. This was manifested by all microrheological parameters studied, i.e., a decrease in MVI and EI and an increase in FI and SLB, although not always statistically significant at *p* < 0.05. The above observations are in line with studies that indicate that US treatment may lead to depolymerisation of hydrocolloids [25]. However, this would result in a noticeable reduction in the viscosity of the product, which is not the case here (as shown in Table 1, changes in the opposite direction), indicating that the phenomena are more complex and ambiguous in interpretation.

**Figure 2:**
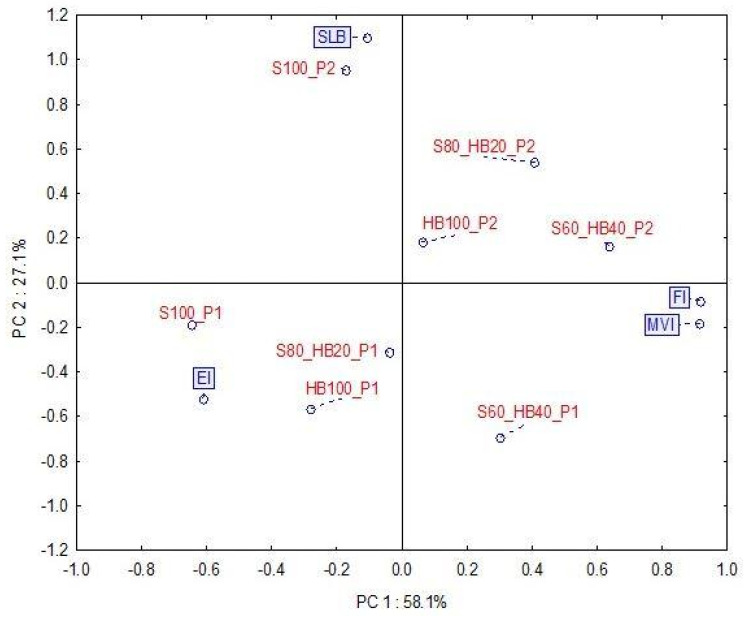
PCA score plot for microrheological properties of haskap berry and strawberry purees. Red labels—samples (P2: produced with ultrasound treatment before pureeing; P1: produced without ultrasound treatment; HB: haskap berry; S: strawberry; followed by the puree proportion 20–100%). Blue labels—microrheological properties: EI—elasticity index [nm^−2^], FI—fluidity index [Hz], SLB—solid–liquid balance [-], and MVI—macroscopic viscosity index [nm^−2^∙s].

To analyse more deeply the relationship between the microrheological properties of the investigated fruit purees, PCA was performed. The PCA data plots represent over 85% of the total variance between samples (Figure 2). The first principal component primarily accounts for discrimination among samples based on their composition, which is largely correlated with variations in sample viscosity. On the other hand, the second principal component differentiates samples based on ultrasound treatment, which can be linked to differences in elastic and viscous behaviour. It should also be noted that S100 puree (regardless of ultrasound treatment) had the most distinct microrheological properties. This also indicates that even a relatively small amount of haskap berry can lead to changes in the microrheology of the puree.

In the case of the S100 and S60_HB40 recipes, samples subjected to US (P2 purees) exhibited a significantly higher FI compared to their untreated counterparts (P1) (Table 2). The SLB significantly increased in all recipes after US, except for the S60_HB40 puree, indicating a transition towards less viscous behaviour. As can be seen in the PCA plot, two groups of samples with similar rheological properties that differ from each other can be identified. Samples S100, S80_HB20, and HB100 produced using method P1 form a group characterised by a lower EI. The group of samples consisting of HB100, S60HB40, and S80_HB20 produced using the P2 method exhibited similar properties due to higher SLB and FI levels. Based on these results, it can be concluded that US treatment causes a reduction in viscosity at zero shear and elasticity on a microscopic scale, as well as an increase in fluidity. These findings confirm the efficacy of US as a texture-modifying technology for fruit purees, consistently reducing viscoelastic properties on the microscale. Furthermore, ultrasound processing can enhance the smoothness and structural stability of the puree, conferring a sensory perception of greater “homogeneity” compared to P1 products, which appear more prone to phase separation. Microrheology, as a relatively new method, is not often used to study the mechanical properties of foods such as fruit juices or purees and has more often been used for testing gels and emulsions [29]. Micro- and macro-rheological results are usually consistent for homogeneous materials, whereas in heterogeneous systems they may vary significantly [30]. Fruit purees are precisely such highly heterogeneous systems, containing both large and small particle fractions. This study confirmed the presence of a wide spectrum of particles and a shift in particle size distribution towards smaller particles after the application of ultrasound, which is directly related to the micro-textural properties of purees. Therefore, when analysing the results of particle motion-based analysis, which measures local viscoelastic properties, it should not be directly translated into the macroscopic properties of puree, as this discrepancy was confirmed by previous studies [31]. As demonstrated in the first part of the experiment, where haskap berry purees were analysed, US treatment can lead to increase in apparent viscosity (Table 1). When analysing the results of microrheological measurements together with the results of apparent viscosity, it should be noted that at this stage it is not possible to fully assess whether the direction of change is positive. On the one hand, an increase in viscosity is positive in terms of product stability and sensory evaluation. On the other hand, the results of the microrheological analysis indicate a shift in the product’s characteristics towards fluid behaviour, which is also important from the point of view of industrial production processes (e.g., pumping and bottling).

#### 2.2.2. Particle Size Distribution

**Figure 3:**
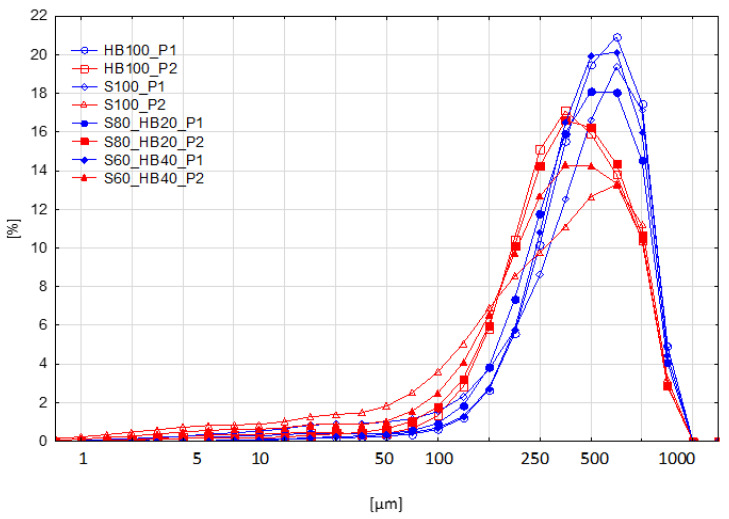
Particle size distribution of haskap berry and strawberry purees produced with ultrasound treatment before pureeing. P1: without ultrasound treatment (blue); P2: with ultrasound treatment (red). HB: haskap berry; S: strawberry; followed by the puree proportion of 20 to 100%.

The particle size distribution was examined in the puree samples (Figure 3). The mode diameter of the distribution, i.e., the particle diameter at which the graph reaches its highest percentage value (corresponding to the most numerous particle fraction), for samples obtained using the P1 method was in the range of 420–550 μm; the exception was S80_HB20_P1, with the mode in the range of 320–420 μm. The modal diameter value for samples obtained using method P2 was in the lower range of 240–320 μm. In this case, S100_P2 was the exception, with a mode diameter at 420–550 μm. For all formulations (except S100), US treatment resulted in a reduction in the mode. The maximum for S100_P1 and S100_P2 falls within the same particle diameter range, but its relative value is significantly lower (from 19.36 ± 0.80% to 13.27 ± 1.97%, respectively).

Table 3 presents the values of particle size parameters (D10, D50, D90, and SPAN) for both production methods (P1 and P2). In order to enable the comparison of data and identification of extreme values, Table 3 was transformed using conditional formatting in the form of a heat map. For each parameter (D10, D50, D90, SPAN), an automatic colour scale was applied, where the colour of the cell corresponds to the numerical value of a given sample within the column. The analysis indicates that samples treated with ultrasound (P2) generally have smaller particles than control samples (P1), as evidenced by significantly lower D10, D50, and D90 values in purees obtained using the P2 method. At the same time, the SPAN for P2 purees was higher, meaning a broader particle size distribution. The ultrasound treatment did not substantially reduce the diameter of the largest particle group but led to an enrichment in small particles, resulting in a broader particle size distribution.

Other authors have similar results, where cavitation and shear forces caused by US led to a reduction in particle size [32,33]. The particle size distribution of control and ultrasound-treated mango nectar [25] is remarkably similar to the results obtained in this study. As in our case, the reduction in the group of large particles was accompanied by an increase in the number of the smallest particles, while no significant changes in SPAN were observed. The observed results generally correspond to the practical application of ultrasound—homogenisation [34]. With appropriately selected parameters, ultrasound waves cause the rupture of cell walls and membranes. It has been proven that the right processing time and frequency can cause the rupture of cells in pathogenic organisms, among others, thus enabling ultrasound to reduce the amount of these organisms in food. For example, in experiments with suspensions of *Escherzchia coli* cells, inactivation was achieved by continuous treatment with ultrasound after several dozen minutes at a frequency of 2 MHz and peak intensities in the range of 2.25–8.7 W/cm^−2^ [35]. In a publication in which ultrasonic homogenisation was applied to aqueous cellulose solutions [36], a significant reduction in particle size was observed, combined with an increase in SPAN, exactly as in the case in the studies discussed here. The most effective treatment parameters were a frequency of 20 kHz for several dozen minutes at a temperature of 40–50 °C, resulting in the greatest reduction in particle size. Both the literature example given and the conclusions from our experience demonstrate the potential of ultrasound to increase the solubility of long-chain carbohydrates.

#### 2.2.3. Sensory Analysis

**Figure 4:**
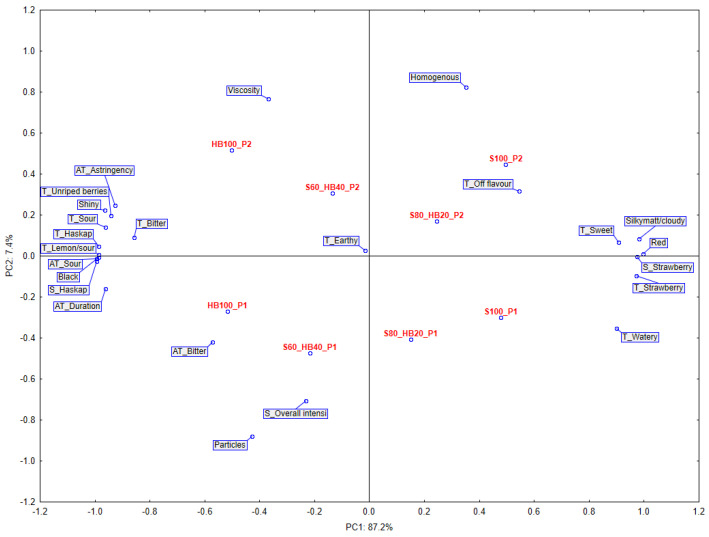
Principal Component Analysis (PCA) from sensory descriptive analysis of haskap berry and strawberry purees produced with ultrasound treatment before pureeing. P1: without ultrasound treatment; P2: with ultrasound treatment. HB: haskap berry; S: strawberry; followed by the puree proportion of 20 to 100%.

The relationships between sensory descriptors (Table 4) and product samples are illustrated in Figure 4. On the basis of the scree plot, two principal components were selected as the optimal number of underlying dimensions, which together accounted for 94.6% of the variation in sensory descriptors of the assessed puree samples. The biplot displays the first and second principal components, which explain 87.2% and 7.4% of the total variance in the sensory data, respectively. The distribution of samples and descriptors along these axes reflects the major trends and discriminative factors observed among the tested purees. Factor 1 (vertical axis) divided the tested samples into two groups with different sensory characteristics as an effect of the recipe of the samples (raw material mixture from which the products were made). The left side of the graph is occupied by samples with 100% or 40% haskap berries (HB100 and S60_HB40). In contrast, the right-hand side of the graph is occupied by samples made mostly from strawberries—S100 and S80_HB20. The arrangement of the samples maintained a logical order—moving from the left to the right side of the graph—the samples reflected the gradual increase in strawberry content. Such a distribution of samples on the plot was associated with all descriptors of taste, appearance, and aftertaste. This confirms the methodological correctness in the composition of samples and the proper training of assessors. Returning to the main objectives of the experiment, it is important to look at the division of groups that Factor 2 evokes (horizontal axis).

All ultrasound-treated samples were projected on the upper side of the graph (P2 samples). Thus, samples where ultrasound was not used were placed on the lower part of the graph (P1 samples). The distribution of samples on the plot was associated mainly with differences in textural characteristics of samples. The descriptors that strongly characterised the P2-labelled samples were, first of all, Viscosity and Homogeneous—both concerning the texture of the sample. On the contrary, the samples labelled P1 were characterised by the presence of fruit particles, as evidenced by the strongly downward-loading descriptor Particles. The Earthy descriptor (not statistically significant) is not interesting from the point of view of the objectives of the study, but interesting for methodological reasons of evaluating the results and the experiment carried out. Its projection in the centre of the PCA graph clearly demonstrates the absence of correlation with the processing method and sample composition. With regard to the evaluation of smell, only three descriptors were used. What can be considered positive is that the descriptors of the characteristic aroma of the fruit used in the samples (strawberry, haskap berry, or a mixture of both) were evaluated in accordance with the actual composition. It can therefore be assumed that the use of ultrasound did not introduce significant changes in the volatile compound composition of the samples (which were not examined instrumentally in this study). Although not statistically significant, it is noteworthy that in three out of four sample pairs the total smell complexity (S_Overall intensity) was judged to be lower in P2 samples. That can suggest that some negative influence of ultrasound treatment can be found on the smell of purees. Similarly, the colour red (characteristic of strawberry puree) and the colour black (characteristic of haskap berry puree) were assessed without difficulty. The descriptors Silkymatt/cloudy and Shiny also acted as opposites and were assessed according to this approach. Consequently, samples with a high proportion of haskap berry (100% and 40%)—were characterised by low Cloudy scores and were seen as highly Shiny. Texture concerning descriptors (Homogeneous, Particle, Viscosity) behaved in a similar and mutually consistent manner. Samples in the P2 group that were treated with ultrasound were assessed as more homogeneous, and their viscosity was rated higher, which is in line with the rheological properties of the products. Unfortunately, for the viscosity parameter (perhaps due to the difficulty of evaluation), the results did not show clear differences, and in the case of samples composed of both fruit species, they were evaluated at a similar level. In the case of basic flavours, only the sour flavour evidently divided the samples into a low-acid group (100 or 80% strawberry) and a high-acid group (100% or 40% haskap berry). Analysis of basic taste scores does not allow any further conclusions to be drawn about the two puree production methods used. The effect of product composition can be seen even more clearly in the case of aftertastes (Astringency, Sour, Duration). For example, assessors discriminated perfectly between samples, and the assessment of aftertaste persistence length (Duration) depended on the level of compounds present in the samples.

Most publications on the effect of ultrasound on sensory quality in fruit processing concern juices. The literature on sensory changes in purees due to ultrasound treatment is extremely limited. Despite the fact that juices are a different product group from purees and that ultrasound is mainly used as a tool to partially replace pasteurisation, comparison of the results obtained with juice seems to be relevant, as the most important thing is to confirm what effects ultrasound has on sensory profiles. On the basis of literature, ultrasound treatment during production of juices and purees generally maintains or improves sensory quality. For example, in a study conducted on orange juice [37], it was proven that the use of ultrasound does not have a negative impact, while also highlighting the strong potential of this technology in food processing as an alternative to thermal processing. In the case of grape juice [38], sensory analysis demonstrated that ultrasound contributed to the improvement of sensory characteristics of the juices in a ranking test in which assessors received four samples (one control sample and three other with different levels of ultrasound treatment). A recent review article [39] emphasises that products processed using US generally have better overall sensory acceptability compared to juices processed conventionally. However, the authors also highlight that sensory properties of products obtained through the use of US are an aspect that is often neglected.

#### 2.2.4. Anthocyanins

The results of the anthocyanin content analysis by the HPLC method are summarised in Table 5. The highest total content of the six anthocyanins was observed in sample HB100_P1 (331.02 ± 12.40 mg 100 g^−1^ FW), which was statistically significantly higher than that in HB100_P2 (319.99 ± 7.67 mg/100 g^−1^ FW). Among all puree samples, only this recipe exhibited a statistically significant effect of the processing method, where ultrasound treatment (P2) resulted in an unfavourable reduction in total anthocyanin content compared to the control process (P1). The lowest total anthocyanin concentrations were found in S100_P1 and S100_P2 samples, at 22.58 ± 0.75 and 22.52 ± 0.24 mg/100 g^−1^ FW, respectively, with pelargonidin-3-glucoside being the dominant compound in these samples. In other purees (S60_HB40 and S80_HB20), anthocyanin levels scaled consistently with the proportion of haskap and strawberry used. For these mixed samples, no statistically significant differences between processing methods were observed for any of the six anthocyanins, with the exception of cyanidin-3-glucoside in S80_HB20_P1 (53.50 ± 0.8 mg/100 g^−1^ FW) and S80_HB20_P2 (51.56 ± 1.05 mg/100 g^−1^ FW), where a significant negative effect was detected. It is worth noting that a 20% addition of haskap berries to strawberry puree resulted in a 3.5-fold increase in anthocyanin concentration compared to pure strawberry puree, while a 40% addition resulted in a 6-fold increase.

In experiments involving the use of ultrasound as a tool to enhance anthocyanin extraction, it has proven to be highly effective [40]. It is worth noting that the ultrasound exposure in this case was 60 min in an ultrasonic bath with a frequency of 40 kHz and a power of 185 W, and no negative effect of treatment time on anthocyanin content was observed. However, it has also been noted that lower US treatment temperatures are favourable for the extraction of anthocyanins, and even temperatures around 50 degrees can negatively affect anthocyanin content [41,42]. However, US treatment of fruit pulp can lead to completely different conclusions, as in our study, where generally no effect of ultrasound on anthocyanin content was observed. The results obtained by other authors are inconsistent in this area, providing both evidence of positive and negative effects of ultrasound. In the case of pulp made from purple tamarillo fruit, US for 10 min resulted in a significant increase in delphinidin-3-rutinoside (+115%) and pelargonidin-3-rutinoside (+270%) content [43]. The results of the experiments conducted depend to a large extent on the choice of methodological parameters. Among generally positive observations of anthocyanin behaviour in experiments attempting to replace traditional pasteurisation with US treatment at elevated temperatures, it has also been reported that ultrasound treatment at 55 °C for 9 min can reduce total anthocyanin content by 7.1% compared to untreated strawberry juice [44]. Additionally, the mechanism of anthocyanin degradation was identified in a model experiment, where a strong correlation was found between the rate of pelargonidin-3-glucoside degradation and the rate of •OH production [45]. This shows that, as in the case of AA degradation, the breakdown of bioactive compounds such as anthocyanins can be caused by the formation of free radicals due to cavitation.

#### 2.2.5. Total Pectin Content

**Figure 5:**
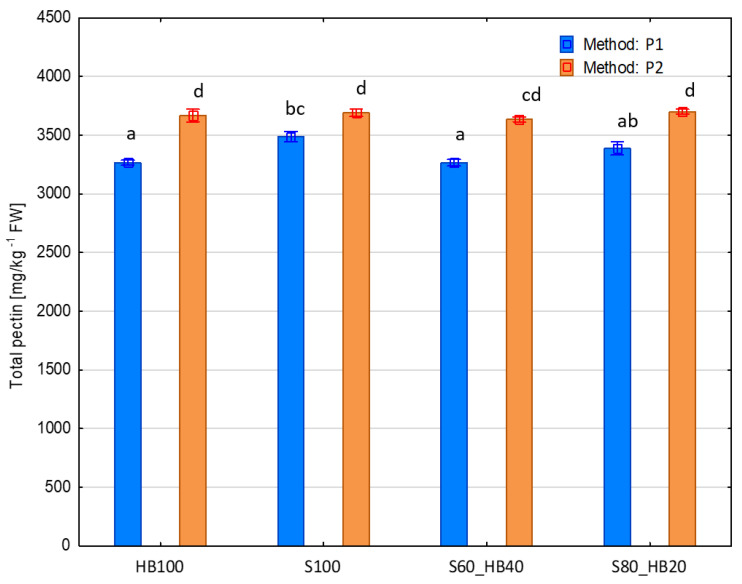
Total pectin content [mg kg^−1^ FW] of haskap berry and strawberry purees produced with ultrasound treatment before pureeing. Mean for samples marked with the same letter do not differ significantly (*p* < 0.05) according to Tukey’s HSD test (*n* = 6). P1: without ultrasound treatment; P2: with ultrasound treatment. HB: haskap berry; S: strawberry; followed by the puree proportion of 20 to 100%.

The pectin content in food products is of great importance for both their quality and nutritional value. In terms of product quality, a higher pectin content results in higher viscosity and density, which are also important for the visual quality of products. If there is a sufficient amount of pectin in the product, there is no separation, i.e., the product is more stable [46]. In terms of nutritional value, the pectin content in food products promotes proper intestinal peristalsis and has a proven beneficial effect on human health [47]. No significant effect of the recipe on the pectin content was found, and its average content for all recipes was 3512.75 ± 174.86 mg kg^−1^ FW (Figure 5). This result is slightly higher than those reported in the literature data, which indicate pectin contents in raw strawberries and haskap berries at levels of 1 to 2% [48,49].

A significant effect of processing method on pectin content was observed in the tested purees (Figure 5). The average total pectin content across all tested samples was 3673.75 ± 25.47 mg kg^−1^ FW. For each recipe, samples obtained with the use of ultrasound were characterised by a statistically significantly higher total pectin content. This is consistent with the fact that ultrasound is a tool for efficient pectin extraction [50].

Pectin content in products is strongly linked to the sensory quality of products, as food consistency is crucial to food perception [51]. Therefore, during sensory evaluation of samples using the descriptive method, the analysis was supplemented with parameters such as Homogeneous, Viscosity, and Particles. PCA was performed to determine the relationship between total pectin content and selected key sensory descriptors for texture and consistency of the purees. Two components (PC1 and PC2) explained 89.7% of the total variance (Figure 4).

**Figure 6:**
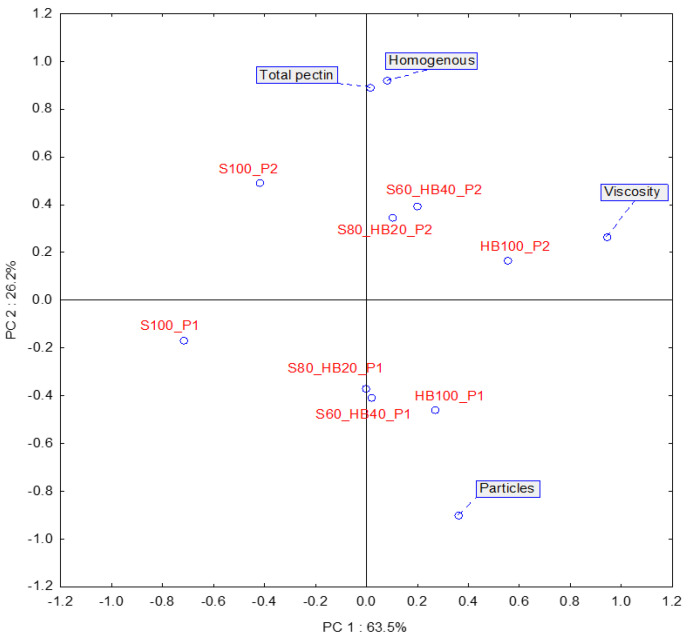
Principal Component Analysis (PCA) from selected sensory descriptors and total pectin content of haskap berry and strawberry purees produced with ultrasound treatment before pureeing. The plot reveals the interrelationship between descriptors (in blue) and samples (in red). P1: without ultrasound treatment; P2: with ultrasound treatment. HB: haskap berry; S: strawberry; followed by the puree proportion of 20 to 100%.

On Figure 6, we can see a visualisation of the analysis with the location of the samples (biplot chart). Based on the location of the descriptors, it can be concluded that the descriptor Viscosity had the greatest impact on the first component (63.5% of the total variance). The upper right quadrant, where this descriptor is located, also includes three purees produced by the P2 method. Sample S100_P2 is located in the upper left quadrant of the graph, which indicates that it was perceived as less viscous despite having a similar pectin content. Sensory evaluation and instrumental measurement often produce different results when assessing food texture and consistency, since human perception is influenced by many factors and interpretation by assessors may not align with objective instrumental data—making the correlation between both approaches not always consistent [52]. In fact, observing the lack of correlation between the measurement methods under study helps to gain knowledge about aspects that are not immediately apparent.

In the case of the second component, explaining 26.2% of the total variance, a high correlation was found between pectin content and the Homogeneous descriptor, and a negative correlation with the Particles descriptor. There is consistency in the data presented here, showing a simultaneous increase in total pectin content and homogeneity.

The presented results concern the impact of US treatment on fruit pulp prior to pureeing. By having access to all the tissues that make up the fruit, acoustic cavitation and shear forces disrupt the cell walls, substantially enhancing the availability and extraction efficiency of pectin. This is well established, as ultrasound application induces intensive breakdown of cellular structures, facilitates the release of pectin, and accelerates both diffusion and penetration phenomena during extraction [50], leading to an increased concentration of pectin in the final product, as it is shown in Figure 5. Although partial degradation of soluble fraction of pectin may also occur [25], the overall pectin yield resulting from structural disintegration, in the case of this study, significantly outweighs this effect.

#### 2.2.6. Organic Acids

**Figure 7:**
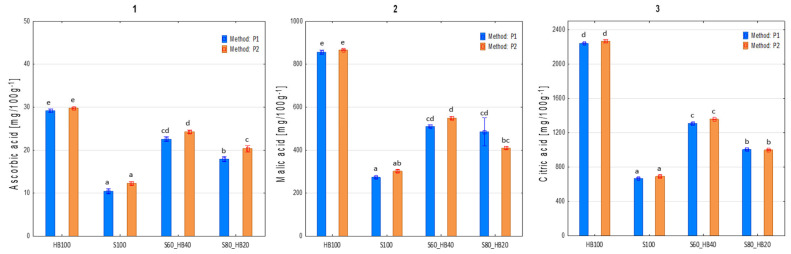
Organic acid content of haskap berry and strawberry purees produced with ultrasound treatment before pureeing. 1—ascorbic acid (AA) (**left**), 2—malic acid (**middle**), 3—citric acid (**right**). Means for samples marked with the same letters do not differ significantly (*p* < 0.05) according to Tukey’s HSD test 705 (*n* = 6). P1: without ultrasound treatment; P2: with ultrasound treatment. HB: haskap berry; S: strawberry; followed by the puree proportion of 20 to 100%.

The content of organic acids and the statistical differences are detailed in Figure 7. Three organic acids—L-ascorbic, malic, and citric acid—were investigated in this study. Technologically, citric and malic acids are valuable components found in fruit raw materials. They not only lower product pH to ensure stability after pasteurisation but also contribute to an appropriate sensory profile. AA is widely considered an important indicator of overall food product quality. As an essential vitamin for humans, the recommended daily intake of AA, according to current European dietary reference values [53], is 80 mg per day. When analysing the impact of processing, the preservation level of AA can serve as an indicator for the proper application of processing methods, such as maintaining appropriate time and temperature regimes. The recipe containing 100% haskap berry (HB_100) showed the highest content of all three tested acids: 29.49 ± 0.74, 861.56 ± 17.12, and 2255.15 ± 36.77 mg/100 g FW for L-ascorbic, malic, and citric acid, respectively. Conversely, the 100% strawberry recipe (S100) contained the lowest levels of these compounds: 11.35 ± 1.47, 287.92 ± 18.54, and 680.26 ± 26.36 mg/100 g FW, for L-ascorbic, malic, and citric acid, respectively. The differences between the contents of all three acids for these two recipes were statistically significant (*p* < 0.05). Logically, the intermediate recipes (S80_HB20 and S60_HB40) contained intermediate acid levels, with a slight increase corresponding to the higher proportion of haskap berries. The processing method impacted the content of these compounds in only a few limited cases. For AA, the type of processing used was statistically significant only in the S80_HB20 sample, where a 12% increase was noted with the P2 method. In all other samples, the differences were not significant, although the acid content was consistently slightly higher with the P2 method.

Based on these data, it can be concluded that the processing method had no significant effect on malic and citric acid content in any of the tested recipes. Ultrasound treatment (P2) applied during the processing of strawberry and haskap berry purees did not significantly affect the content of malic and citric acid but may have had a beneficial effect on the content of AA. In some cases, observations by other authors suggest opposite results. In the case of raspberry and blueberry purees, the use of ultrasound had a degrading effect on AA, causing a 30% reduction in the content of this compound [17]. Similarly, studies using tomato puree have shown an increase in ascorbate peroxidase activity, resulting in a decrease in AA concentration [54]. Ascorbate peroxidase is an enzyme involved in the decomposition of hydrogen peroxide using AA as an electron donor, resulting in oxidation and a consequent reduction in puree products. The activity of this enzyme during storage or processing of tomato puree promotes the degradation of AA, significantly reducing its content in the final product. When comparing these results with our study, it should be noted that ultrasound in those cases was applied to the material without heat treatment; thus, ascorbate peroxidase remained active. On the contrary, in our experiment, the product was heat-treated before treatment, which proved to be a better solution in terms of AA content, possibly due to the inactivation of enzymes responsible for the degradation of AA. In experiments with fruit and vegetable juices (orange, sweet lime, carrot, and spinach juices), similar results were obtained even when treating the material before heat treatment. In some cases, ultrasound treatment proved to be significantly superior to thermal pasteurisation in terms of AA content [19]. The authors attributed this effect as being closely related to the temperature of the pasteurisation process itself, as temperature is the main cause of AA depletion [55].

However, the results obtained from other studies do not always support this conclusion. One example is a comprehensive study on ultrasound treatment of red raspberry puree and AA solutions (used as a model system), which systematically investigated the effect of varying ultrasound frequencies and power over 10 min intervals of sample collection [56]. That study paid particular attention to temperature increases during sonication and demonstrated that AA degradation is influenced not only by thermal effects, but also by the formation of free radicals induced by acoustic cavitation. Although the mechanism of AA degradation during cavitation is not yet fully understood, research suggests that two main effects (thermolysis and reaction with free radicals) are the most likely causes of AA degradation during ultrasound treatment [57].

## 3. Materials and Methods

### 3.1. Plant Material

The raw material used in the experiment came from commercial cultivation and was purchased during the 2024 harvest season from local producers. The experiment was conducted on two fruit species: strawberries (*Fragaria × ananassa* cv. ‘Florence’) and haskap berry (*Lonicera caerulea* L. cv. ‘Wojtek’). Immediately after transport, the fruits were washed, sorted, and frozen at −25 °C until processing.

### 3.2. Preparation of Samples—Experiment Plan

**Figure 8:**
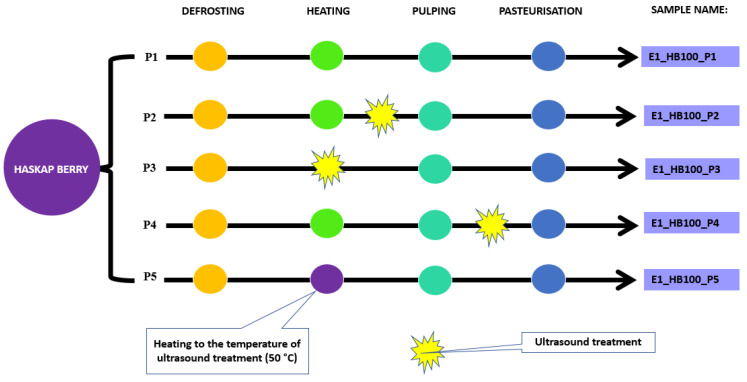
Diagram of “stage I” of the experiment with ultrasound treatment in different sequences of haskap berry puree production.

The experiment was conducted in two stages. In the first one, it was investigated which of the proposed technological schemes (Figure 8) for obtaining puree with ultrasound would be optimal. Ultrasound (US) was used as an additional treatment before pulping (P2), after pulping (P4), or as an alternative to heating the raw material (P3). In evaluating the combinations, the yield of puree (as a percentage of the raw material), viscosity, total anthocyanin content, and total polyphenol content were taken into account.

In the first stage of the experiment, haskap berry fruits (without additives) were used as raw material, which correspond to the aim of the work—to increase the content of bioactive compounds in the final product by ultrasound treatment. Samples labelled P1 and P5 were control objects (produced without ultrasound) against which the results from the other samples were compared. The sample labelled P5 was an additional control object, produced without ultrasound but heated to the same temperature as that generated by US. Since we observed increased temperatures upon ultrasound treatment, this control negates that effect by matching the heating temperature to the temperature observed during ultrasound treatment in P2, P3, and P4.

Ultrasonic treatment (UT) (P2–P4) was carried out on 1000 g fruit samples for 10 min. The samples were treated in an ultrasonic homogeniser at a frequency of 20 KHz (Cole-Palmer Instrumental Company, VCX-750, Vernon Hills, IL, USA) equipped with a 13 mm diameter probe. The device operated at a constant amplitude of 80%, using the maximum power of the device (750 W). The probe tip was immersed in the fruit pulp at the geometric centre of the container, 10 cm below the surface.

Heating of fruits to 80 °C (P1, P2, P4), as well as heating to 50 °C (P5), was carried out in a metal pot on an electrical cooktop. In order to enable ultrasound treatment, pre-shredding was necessary for combinations in which heating to 80 °C was not used. Pre-shredding of frozen fruit (objects P3 and P5) was carried out in a Thermomix (250 g portions, 5 s). Three technological repetitions were performed for each production scheme (P1–P5) in stage I.

**Figure 9:**
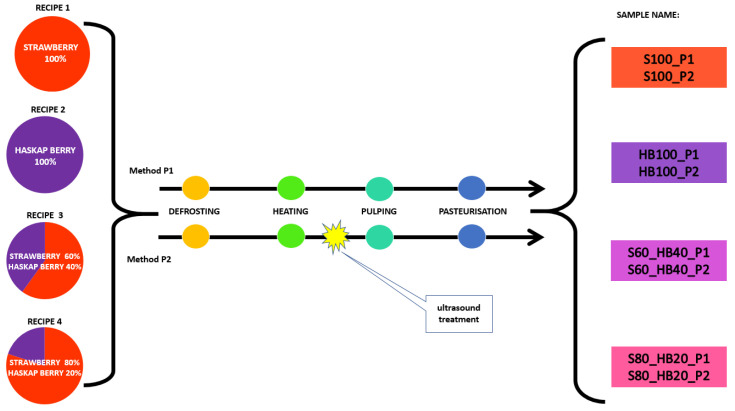
Diagram of “stage II” of the experiment with ultrasound treatment during haskap and strawberry purees production with four different recipes.

All purees samples prepared according to schemes P1–P5 were pasteurised at 90 °C for 20 min, then cooled and analysed. After verifying the results of the analyses of the purees from stage I, combination P2 was selected to carry out stage II, (see Figure 9 for a diagram of the experimental scheme of stage II). The P1 method was used as the control. In this part of the experiment, the selected processing methods were tested on four recipes: strawberry—100% (S100); haskap berry—100% (HB100); strawberry—80% + haskap berry—20% (S80_HB20); strawberry—60% + haskap berry—40% (S60_HB40). After making purees for the four recipes according to schemes P1 and P2, all analyses were performed immediately. Three technological repetitions were performed for each puree recipe.

### 3.3. Physico-Chemical Analysis

#### 3.3.1. Apparent Viscosity

Viscosity was determined using a Brookfield LVDVII viscometer (Brookfield Eng., Middleboro, MA, USA), following the method initially proposed by Jenni et al. [58]. Measurements were performed at a constant spindle speed of 60 rpm and a sample temperature of 20 °C. Before analysis, the samples were transferred into 100 mL glass beakers and equilibrated at 20 °C until a constant temperature was reached (approximately 2 h). During incubation, the beakers were covered with lids to minimise evaporation and prevent the formation of a dried layer on the surface. The Brookfield spindle was then immersed in the sample (in the centre of the beaker) to the depth marked on the spindle and operated at the set rotational speed. Apparent viscosity of the puree was expressed in mPa·s.

#### 3.3.2. Total Anthocyanins Content (TAC)

Total anthocyanin content was determined by the pH differential method described by Giusti and Wrolstad [59], using a Cary 3000 Bio UV–visible spectrophotometer (Varian, Melbourne, Australia). Anthocyanin content was obtained using the standard pH differential equation and the final values were normalised to the sample mass. Anthocyanin concentrations were calculated assuming cyanidin-3-glucoside as the reference compound, with a molar absorptivity of ε = 28,800 L·mol^−1^·cm^−1^ and a path length of 1 cm. Results were expressed as mg cyanidin-3-glucoside equivalents per 100 g fresh weight of puree.

#### 3.3.3. Total Polyphenol Content (TPC)

Total polyphenol content was measured spectrophotometrically following the method initially proposed by Singleton and Rossi [60]. A total of 10 g of samples were homogenised with 70% ethanol and centrifuged for 10 min at 20,000 rpm. An aliquot of 0.4 mL supernatant was mixed with 1.6 mL sodium carbonate solution (7.5% *w*/*v*), followed by the addition of 2 mL Folin–Ciocalteu phenol reagent diluted with distilled water 1:10, was added. Incubation was carried out for 30 min at ambient temperature in the absence of light. Absorbance was measured at a wavelength of 765 nm, and polyphenol content was expressed as gallic acid equivalents per 100 g^−1^ fresh weight of puree.

#### 3.3.4. Microrheological Properties

Microrheological properties of the purees were examined using diffusing wave spectroscopy (DWS) with a Rheolaser Master Lab 6 device (Formulaction, Toulouse, France). Measurements were performed in full characterisation mode using 20 mL glass vials, ensuring the absence of air bubbles. Samples were analysed at ambient temperature controlled by the device’s built-in thermostatic system. During the measurement, the relationships between the correlation functions of scattered light intensity over time were recorded, and the microrheological parameters describing the behaviour of the system were determined from them. The following parameters were determined: elasticity index (EI; nm^−2^), solid–liquid balance (SLB), macroscopic viscosity index (MVI; nm^−2^·s), and fluidity index (FI; Hz), which describe the proportion of elastic and viscous components and the mobility of particles in the tested samples.

#### 3.3.5. Particle Size Distribution

Particle size distribution was analysed by laser diffraction method (Bettersizer S3, Dandong, China). Measurement time was 30 s, and particle size was calculated based on Mie theory. The instrument is equipped with a stirrer to prevent sedimentation of the particles by circulating the water solution of the sample through the measuring system. The result of the analysis is presented as a percentage distribution of individual particle fractions. Based on D10, D50, and D90 particle distribution parameters, span was calculated according to equation:

SPAN = (D90 − D10)/D50(1)

where

SPAN—width or broadness of a particle size distribution;
D90—the particle size at which 90% of the particles are smaller than this value;
D10—the particle size at which 10% of the particles are smaller than this value;
D50—the median particle size, where 50% of the particles are smaller and 50% are larger.

#### 3.3.6. Sensory Analysis

Descriptive sensory analysis was performed by a panel of ten trained panellists (eight women, mean age 31.4 years). Panellists were selected and trained according to ISO guidelines (ISO 8586-2, International Standard Organisation, 2023) [61]. Sensory sessions were conducted at the University of Copenhagen, Department of Food Science. Sensory facilities complied with ISO guidelines for the design of test rooms (ISO 8589, International Standard Organisation, 2007) [62]. Evaluation sessions took place in a sensory laboratory. Samples were labelled with random three-digit codes. A 15 cm unstructured scale with relevant end-point anchors was used for all descriptors. Samples were poured in 60 mL cups and stored at 20 °C for at least one hour before serving. Data were collected digitally with a software dedicated for sensory assessment (Fizz, Biosystemes, Couternon, France). For palate-cleansing, room temperature water and plain crackers were used. Three tasting replicates were conducted in separate sessions. In the first stage of sensory training, panel members were asked to taste the provided puree samples and consider how they would describe their appearance, aroma, and taste. To facilitate this task, the panellists were given a list of terms prepared by the laboratory staff, with the clarification that they could reject any of the proposed terms or suggest additional ones. Particular attention was paid to the degree of agreement between panellists in the selection and acceptance of individual sensory descriptors. A teference list (Table 3) was also delivered by scientific staff, followed by a discussion stage with the assessors. In the second stage, the panellists were trained for the assessment of the puree samples. Twenty-four descriptors were chosen for the final evaluation belonging to four categories: appearance, smell, texture, taste, and aftertaste.

#### 3.3.7. Pectin

Total pectin content was determined according to the spectrophotometric method with carbazole in a sulfuric acid medium, as described by Taylor et al. [63]. One gram of puree was extracted with hot 95% ethanol (85 °C) to remove soluble sugars and washed with 63% ethanol. The alcohol-insoluble residue was treated with 10 mL of 1 N NaOH for 15 min, and the extract was filtered. A total of 1 mL of the extract was reacted with 0.5 mL of a 0.1% alcoholic solution of carbazole and 6 mL of concentrated H_2_SO_4_ heated at 85 °C for 5 min, and after cooling, the absorbance was measured at 525 nm. Pectin content was calculated from a galacturonic acid calibration curve and expressed as mg d-(+)-galacturonic acid per kg fresh weight of puree.

#### 3.3.8. HPLC Analysis of Anthocyanins

The analysis of anthocyanin content was conducted in accordance with the method described by Mieszczakowska-Frąc et al. [64], using high-performance liquid chromatography with an Agilent 1200 system (Agilent Technologies, Santa Clara, CA, USA) equipped with a diode array detector. Separation was performed using a Phenomenex^®^ Fusion RP column (250 mm × 4.6 mm, 4 µm; Phenomenex, Torrance, CA, USA). Gradient elution was applied at a flow rate of 1 mL min^−1^ and a column temperature of 25 °C, with eluent 1 consisting of water and formic acid (95:5, *v/v*) and eluent 2 consisting of acetonitrile. Anthocyanins were detected at a wavelength of 520 nm. Extractions were carried out from 5 g of puree homogenised in 70% methanol (JT Baker Chemicals, Phillipsburg, NJ, USA), and the supernatant prior to analysis was filtered through a 0.45 µm syringe filter. Quantification of anthocyanins was performed using external calibration curves prepared for each anthocyanin standard. Anthocyanin content was expressed as mg 100 g^−1^ fresh weight of puree.

#### 3.3.9. HPLC Analysis of L-Ascorbic, Malic, and Citric Acids

The content of L-ascorbic, malic, and citric acids was analysed using high-performance liquid chromatography with an Agilent 1200 (Agilent Technologies, Santa Clara, CA, USA) with a diode array detector. An SUPELCOSIL LC-18 reversed-phase column (C18, 250 × 4.6 mm, 5 µm; Supelco, Bellefonte, PA, USA) with a pre-column was used. A 1% KH_2_PO_4_ solution adjusted to pH 2.5 served as the mobile phase at a flow rate of 0.8 mL min^−1^ (isocratic elution) and the column temperature was set at 30 °C. Detection of L-ascorbic acid (AA) was performed at 244 nm, whereas malic and citric acids were monitored at 210 nm. Acids were extracted by homogenising 2.5 g of puree in a 6% HPO_3_ solution, after which the supernatant was filtered through filter paper. Quantification of acids was carried out using external calibration curves prepared for each standard. The results were expressed as mg per 100 g^−1^ fresh weight of puree.

#### 3.3.10. Statistical Analysis of Data from “Stage I” of the Experiment

The results obtained were analysed in a completely randomised design using one-way analysis of variance (ANOVA), separately for each parameter analysed. To compare the mean values of apparent viscosity, process yield, total polyphenol content (TPC), and total anthocyanin content (TAC) in purees, Tukey’s HSD test was used as a post hoc test. Differences between means were considered statistically significant at a significance level of *p* < 0.05. Statistical analyses were performed using STATISTICA 13 (Dell Inc., Tulsa, OK, USA).

#### 3.3.11. Statistical Analysis of Data from “Stage II” of the Experiment

Statistical analysis was performed with the use of STATISTICA 13 (Dell Inc., Tulsa, OK, USA). Treatments were analysed in a completely randomised design using two-way ANOVA. Tukey’s HSD test was selected for the comparison of particle size distribution, pectin content, HPLC results for anthocyanins and organic acids in purees. The comparison was performed at a significance level of *p* < 0.05.

Data from descriptive sensory analysis and microrheological properties analysis were analysed in STATISTICA 13 software (Dell Inc., Tulsa, OK, USA). To evaluate major patterns among samples and product characteristics and to reduce the dimensionality of the dataset, Principal Component Analysis (PCA) was applied. Results were visualised on two-dimensional biplots, enabling interpretation of similarities and differences among the projected samples.

## 4. Conclusions

The study demonstrated that the application of US treatment as an additional treatment prior to pureeing effectively enhanced both the yield and apparent viscosity of the obtained purees. Analysis indicated that US treatment influenced the microrheological properties, shifting their characteristics toward more fluid-like behaviour. These structural modifications were closely related to particle size reduction and pectin interactions, which collectively contributed to the observed rheological changes, though not always in the expected direction. Particle size distribution analysis confirmed that US treatment produced smaller particles but resulted in a broader size distribution, whereas sensory evaluation combined with Principal Component Analysis (PCA) distinguished US-treated samples from untreated ones. Purees processed with US received higher scores for the descriptors Homogeneous and Viscosity. Regardless of the recipe, pectin content increased following US treatment. However, sensory perception differed substantially from the measured pectin content, suggesting that production technology and raw material characteristics play a decisive role in shaping the final product properties. US application during the processing of strawberry and haskap berry purees did not significantly affect malic or citric acid content but had a beneficial influence on L-ascorbic acid levels. A decrease in total anthocyanin content was observed in selected samples, suggesting that compound stability during ultrasound exposure depends strongly on the characteristics of the medium and the chemical nature of individual compounds. Comparison with literature data proved challenging due to the scarcity of studies investigating ultrasound application prior to pureeing of fruit pulp. Most published works focus on ultrasound-assisted juice extraction, microbial stabilisation, or texture modification. Thus, this research represents, to the best of the authors’ knowledge, the first detailed analysis of US treatment effects on the sensory and physico-chemical characteristics of berry purees when used as an additional treatment before pureeing.

Nevertheless, several limitations of the processing scheme should be emphasised. Efficient propagation of ultrasonic waves requires a sufficient liquid phase, whereas fruit pulps typically exhibit high viscosity and a dense structure. The low dry matter content of strawberries and haskap berries enabled the effective use of US in this study; however, this approach may not be universally applicable to other fruits. Although dilution of raw material has been proposed in some studies to facilitate sonication, such practice alters product composition and is thus not recommended for high-quality or clean-label products. Furthermore, combining ultrasound with thermal treatment compromises its non-thermal character; therefore, temperature input should be minimised during implementation.

Although ultrasound treatment did not enhance the extraction of anthocyanins from berry skins, it demonstrated a marked influence on the rheological behaviour and processing efficiency of berry purees. The observed shift toward more liquid-like microrheological behaviour, accompanied by increased apparent viscosity, highlights ultrasound’s potential to modify flow properties and improve processing conditions in industrial environments.

Further research should focus on the possibility of repeating the observations on a larger scale and implementing the technology in an industrial system, as the mechanism of action of ultrasound, the effectiveness and safety of the technology, are already well recognised.

## Figures and Tables

**Table 1:** Apparent viscosity [mPa·s] of the haskap berry puree using ultrasound.

Sample	Apparent Viscosity [mPa·s]
E1_HB100_P1	698 ± 82 c
E1_HB100_P2	1108 ± 100 d
E1_HB100_P3	185 ± 15 a
E1_HB100_P4	335 ± 43 b
E1_HB100_P5	170 ± 22 a

P1: puree without ultrasound treatment; P2: ultrasound treatment before pureeing; P3: ultrasound treatment before pureeing, without heating; P4: ultrasound treatment after pureeing; and P5: puree heated to match the temperature reached during ultrasound processing. Values are expressed as mean ± SD (*n* = 6). Different letters indicate statistically significant differences between samples (*p* < 0.05) according to HSD Tukey’s test.

**Table 2:** Microrheological properties of haskap berry and strawberry purees produced with ultrasound treatment before pureeing.

Sample	Elasticity Index (nm^−2^)	Fluidity Index (Hz)	Solid–Liquid Balance (-)	Microscopic Viscosity Index (nm^−2^∙s)
HB100_P1	0.0017 ± 0.0001 ab	0.72 ± 0.29 a	0.52 ± 0.00 a	0.0050 ± 0.0021 c
HB100_P2	0.0013 ± 0.0001 a	2.84 ± 1.77 ab	0.60 ± 0 03 bc	0.0010 ± 0.0005 abc
S100_P1	0.0030 ± 0.0004 c	0.94 ± 0.58 a	0.62 ± 0.02 bc	0.0035 ± 0.0019 abc
S100_P2	0.0017 ± 0.0000 ab	5.32 ± 0.25 b	0.72 ± 0.01 d	0.0004 ± 0.0000 a
S60_HB40_P1	0.0021 ± 0.0003 b	0.80 ± 0.31 a	0.55 ± 0.03 ab	0.0047 ± 0.0024 bc
S60_HB40_P2	0.0014 ± 0.0001 a	3.89 ± 2.04 ab	0.63 ± 0.06 bc	0.0008 ± 0.0007 ab
S80_HB20_P1	0.0023 ± 0.0004 b	1.01 ± 0.51 a	0.59 ± 0.01 ab	0.0035 ± 0.0012 abc
S80_HB20_P2	0.0015 ± 0.0001 ab	5.13 ± 1.39 b	0.68 ± 0 01 cd	0.0004 ± 0.0001 a

P1: without ultrasound treatment; P2: with ultrasound treatment. HB: haskap berry; S: strawberry; followed by the puree proportion of 20 to 100%. Means for samples marked with the same letters do not differ significantly (*p* < 0.05) according to Tukey’s HSD test (*n* = 6).

**Table 3:** Particle size distribution parameters (D10, D50, D90, and SPAN) of haskap berry and strawberry purees produced with ultrasound treatment before pureeing. P1: without ultrasound treatment; P2: with ultrasound treatment. HB: haskap berry; S: strawberry; followed by the puree proportion of 20 to 100%.

SAMPLE	D10	D50	D90	SPAN
HB100_P1	176.77 ± 4.03 f	382.68 ± 10.64 d	651.1 ± 10.75 cb	1.24 ± 0.02 a
HB100_P2	119.48 ± 1.22 d	286.85 ± 7.5 ab	586.28 ± 10.42 a	1.63 ± 0.03 c
S100_P1	95.09 ± 8.56 c	366.38 ± 16.83 cd	649.87 ± 13.68 cb	1.52 ± 0.05 c
S100_P2	36.21 ± 4.09 a	257.57 ± 31.94 a	592.67 ± 42.32 ab	2.17 ± 0.14 e
S80_HB20_P1	142.23 ± 2.97 e	346.52 ± 25.66 c	622.88 ± 48.64 ab	1.38 ± 0.04 b
S80_HB20_P2	116.62 ± 5.02 d	292.02 ± 11.07 b	586.52 ± 25.7 a	1.61 ± 0.05 c
S60_HB40_P1	171.95 ± 4.9 f	369.47 ± 10.51 cd	638.75 ± 15.46 cb	1.26 ± 0.02 a
S60_HB40_P2	71.24 ± 4.48 b	270.67 ± 9.84 ab	587.52 ± 12.9 a	1.91 ± 0.05 d

The lowest values in the column are marked in red, intermediate values in yellow, and the highest values in green. Values are expressed as mean ± SD (*n* = 6). Different letters indicate statistically significant differences between samples processed without (P1) and with ultrasound (P2), (*p* < 0.05) according to Tukey’s HSD test. P1: without ultrasound treatment; P2: with ultrasound treatment. HB: haskap berry; S: strawberry; followed by the puree proportion of 20 to 100%.

**Table 4:** Applied sensory descriptors for analysis of haskap berry and strawberry purees produced with ultrasound treatment before pureeing.

No.	Descriptor	Definition	Abbreviation	Reference Material
Smell (Intensity when sniffed)				
1	Overall Intensity	Overall complicity of the smell of the sample	S_Overall Intensity	-
2	Strawberry	Specific smell of strawberry	S_Strawberry	Boiled strawberry
3	Haskap	Specific smell of haskap berry	S_Haskapberry	Sample HB_100
Taste (Intensity when tasted)				
4	Sour	Basic taste—overall sour	T_Sour	Solution: 1.2 g citric acid/1 L water
5	Sweet	Basic taste—overall sweet	T_Sweet	Solution: 12 g sucrose/1 L water
6	Bitter	Basic taste—overall bitter	T_Bitter	Solution: 0.54 g caffeine/1 L water
7	Strawberry	Taste of strawberry	T_Strawberry	Fresh strawberry fruit
8	Haskap	Taste of haskap berry	T_Haskapberry	Sample HB100
9	Lemon/sour	Taste of lemon sourness	T_LemonSour	Lemon juice freshly pressed
10	Watery	A watery flavour (diluted)	T_Watery	-
11	Earthy	A flavour of soil	T_Earthy	Fresh beetroot, grated
12	Unripe berries	Specific taste of unripe berries	T_Unripe	Mixture of unripe berries: red currant/black currant/raspberry
13	Off flavour	Foreign flavour, not typical for fruit preserves	T_Off flavour	-
Aftertaste (Counting 1–5 and then evaluating aftertaste)				
14	Sour aftertaste	Overall sour aftertaste-	AT_Sour	-
15	Bitter aftertaste	Overall bitter aftertaste	AT_Bitter	-
16	Astringent	Dry sensation on teeth, when moving tongue over after ingestion	AT_Astringent	Chokeberry juice (purchased on the market)
17	Duration of aftertaste	Length of flavour persistence in the mouth within 10 s	AT_Duration	-
Other modalities				
18	Shiny	Concern surface, degree of shine	Shiny	Sample HB100_P1
19	Silky matt/cloudy	Concern surface, how much it is silky-matt and cloudy	Silkymatt/cloudy	Sample S100_P2
20	Particles	Look at the back of the spoon and count the number of particles	Particles	-
21	Homogeneous	Visual assessment of the degree of unevenness in the sample structure	Homogeneous	-
22	Viscosity	Take a sample with a spoon and pour it back into the container—assess the viscosity visually	Viscosity	-
Appearance				
23	Colour: black	Compared to printed references	Black	Colour—RAL 9005
24	Colour: red	Compared to printed references	Red	Colour—RAL 0104040

HB100_P1: haskap berry puree without ultrasound treatment; S100_P2: strawberry puree with ultrasound treatment.

**Table 5:** Anthocyanin content of haskap berry and strawberry purees produced with ultrasound treatment before pureeing.

Sample	Method	Cyanidin-3-Glucoside	Cyanidin-3-Malonylglucoside	Cyanidin-3-Rutinoside	Cyanidin-3,5-Diglucoside	Pelargonidin-3-Glucoside	Peonidin-3-Glucoside	Total Anthocyanins
		[mg/100 g]						
HB100	P1	282.31 ± 10.34 a	0 ± a	24.06 ± 0.84 a	15.05 ± 0.57 a	2.64 ± 0.15 a	6.96 ± 0.64 a	331.02 ± 12.4 a
	P2	272.87 ± 5.78 a	0 ± a	23.37 ± 0.63 a	14.5 ± 0.65 a	2.55 ± 0.29 a	6.71 ± 0.63 a	319.99 ± 7.67 a
S100	P1	1.1 ± 0.21 a	0.35 ± 0.03 a	0 ± a	0 ± a	21.13 ± 0.62 a	0 ± a	22.58 ± 0.75 a
	P2	1.38 ± 0.13 a	0.39 ± 0.06 a	0 ± a	0 ± a	20.74 ± 0.16 a	0 ± a	22.52 ± 0.24 a
S60_HB40	P1	105.98 ± 3.34 a	0.47 ± 0.05 b	8.99 ± 0.19 a	5.94 ± 0.2 a	15.57 ± 0.75 a	2.67 ± 0.16 a	139.62 ± 2.82 a
	P2	100.69 ± 1.13 b	0.55 ± 0.02 a	8.64 ± 0.17 b	6.04 ± 0.14 a	14.92 ± 0.42 a	2.55 ± 0.19 a	133.4 ± 1.26 b
S80_HB20	P1	53.5 ± 0.8 a	0.26 ± 0.2 a	4.78 ± 0.08 a	3.23 ± 0.35 a	18.9 ± 0.98 a	1.49 ± 0.06 a	82.17 ± 1.25 a
	P2	51.56 ± 1.05 b	0.37 ± 0.19 a	4.66 ± 0.21 a	3.28 ± 0.13 a	18.11 ± 0.61 a	1.54 ± 0.16 a	79.52 ± 1.16 b

P1: without ultrasound treatment; P2: with ultrasound treatment. HB: haskap berry; S: strawberry; followed by the puree proportion of 20 to 100%. Means for samples marked with the same letters do not differ significantly (*p* < 0.05) according to Tukey’s HSD test (*n* = 6).

## Data Availability

The original contributions presented in this study are included in the article. Further inquiries can be directed to the corresponding author.

## Abbreviations

The following abbreviations are used in this manuscript:

S	Strawberry
HB	Haskap Berry
US	Ultrasound Treatment
AA	Ascorbic Acid
TPC	Total Polyphenol Content
TAC	Total Anthocyanin Content
EI	Elasticity Index
FI	Fluidity Index
SLB	Solid–Liquid Balance
MVI	Macroscopic Viscosity Index
PCA	Principal Component Analysis
FW	Fresh Weight

## References

[1] Molina A.K., Vega E.N., Pereira C., Dias M.I., Heleno S.A., Rodrigues P., Fernandes I.P., Barreiro M.F., Kostić M., Soković M. (2019). Promising Antioxidant and Antimicrobial Food Colourants from *Lonicera caerulea* L. var. kamtschatica. Antioxidants.

[2] Newerli-Guz J., Śmiechowska M., Drzewiecka A., Tylingo R. (2023). Bioactive Ingredients with Health-Promoting Properties of Strawberry Fruit (*Fragaria* x *Ananassa* Duchesne). Molecules.

[3] Golovinskaia O., Wang C.-K. (2021). Review of Functional and Pharmacological Activities of Berries. Molecules.

[4] Bader Ul Ain H., Tufail T., Javed M., Tufail T., Arshad M.U., Hussain M., Gull Khan S., Bashir S., Al Jbawi E., Abdulaali Saewan S. (2022). Phytochemical Profile and Pro-Healthy Properties of Berries. Int. J. Food Prop..

[5] JBT Corporation (2021). Technologies for Processing Value-Added Berry Fruit Products.

[6] Nawirska-Olszańska A., Biesiada A., Sokół-Łętowska A., Kucharska A.Z. (2016). Effect of Preparation and Storage Conditions on Physical and Chemical Properties of Puree, Puree Juices and Cloudy Juices Obtained from Pumpkin with Added Japanese Quince and Strawberries. Not. Bot. Horti Agrobot. Cluj-Napoca.

[7] Aguilera J.M. (2024). Berries as Foods: Processing, Products, and Health Implications. Annu. Rev. Food Sci. Technol..

[8] Al-Juhaimi F., Ghafoor K., Özcan M.M., Jahurul M.H.A., Babiker E.E., Jinap S., Sahena F., Sharifudin M.S., Zaidul I.S.M. (2018). Effect of Various Food Processing and Handling Methods on Preservation of Natural Antioxidants in Fruits and Vegetables. J. Food Sci. Technol..

[9] Chemat F., Rombaut N., Sicaire A.-G., Meullemiestre A., Fabiano-Tixier A.-S., Abert-Vian M. (2017). Ultrasound Assisted Extraction of Food and Natural Products. Mechanisms, Techniques, Combinations, Protocols and Applications. A Review. Ultrason. Sonochem..

[10] Zhu X., Das R.S., Bhavya M.L., Garcia-Vaquero M., Tiwari B.K. (2024). Acoustic Cavitation for Agri-Food Applications: Mechanism of Action, Design of New Systems, Challenges and Strategies for Scale-Up. Ultrason. Sonochem..

[11] McHugh T. (2016). Putting Ultrasound to Use in Food Processing. Food Technol..

[12] Chen L., Chen L., Zhu K., Bi X., Xing Y., Che Z. (2020). The Effect of High-Power Ultrasound on the Rheological Properties of Strawberry Pulp. Ultrason. Sonochem..

[13] Mahmoud M., Alelyani M., Ahmed A.M., Fagiry M.A., Alonazi B., Abdelbasset W.K., Davidson R., Osman H., Khandaker M.U., Musa M.A. (2023). Ultrasonic Technology as a Non-Thermal Approach for Processing of Fruit and Vegetable Juices: A Review. Int. J. Food Prop..

[14] Bhargava N., Mor R.S., Kumar K., Sharanagat V.S. (2020). Advances in Application of Ultrasound in Food Processing: A Review. Ultrason. Sonochem..

[15] Tsikrika K., Chu B., Bremner D.H., Lemos A. (2022). Effect of Ultrasonic Treatment on Enzyme Activity and Bioactives of Strawberry Puree. Int. J. Food Sci. Amp Technol..

[16] Wu W., Ma X., Wang Y., Yu Y., Huo J., Huang D., Sui X., Zhang Y. (2025). Amplifying Bioactivity of Blue Honeysuckle (*Lonicera caerulea* L.) Fruit Puree through Ultrasonication: Antioxidant and Antiproliferative Activity. Ultrason. Sonochem..

[17] Medina-Meza I.G., Boioli P., Barbosa-Cánovas G.V. (2016). Assessment of the Effects of Ultrasonics and Pulsed Electric Fields on Nutritional and Rheological Properties of Raspberry and Blueberry Purees. Food Bioprocess Technol..

[18] Guimarães J.T., Silva E.K., Ranadheera C.S., Moraes J., Raices R.S.L., Silva M.C., Ferreira M.S., Freitas M.Q., Meireles M.A.A., Cruz A.G. (2019). Effect of High-Intensity Ultrasound on the Nutritional Profile and Volatile Compounds of a Prebiotic Soursop Whey Beverage. Ultrason. Sonochem..

[19] Khandpur P., Gogate P.R. (2015). Effect of Novel Ultrasound Based Processing on the Nutrition Quality of Different Fruit and Vegetable Juices. Ultrason. Sonochem..

[20] Keenan D.F., Tiwari B.K., Patras A., Gormley R., Butler F., Brunton N.P. (2012). Effect of Sonication on the Bioactive, Quality and Rheological Characteristics of Fruit Smoothies. Int. J. Food Sci. Amp Technol..

[21] Nikkhah E., Khaiamy M., Heidary R., Azar A.S. (2010). The Effect of Ascorbic Acid and H_2O_2 Treatment on the Stability of Anthocyanin Pigments in Berries. Turk. J. Biol..

[22] Chua L.S., Thong H.Y., Soo J. (2024). Effect of pH on the Extraction and Stability of Anthocyanins from Jaboticaba Berries. Food Chem. Adv..

[23] Queiroz F. (2020). Optimization for Sensory and Nutritional Quality of a Mixed Berry Fruit Juice Elaborated with Coconut Water. Food Sci. Technol..

[24] Wendin K., Ekman S., Bülow M., Ekberg O., Johansson D., Rothenberg E., Stading M. (2010). Objective and Quantitative Definitions of Modified Food Textures Based on Sensory and Rheological Methodology. Food Nutr. Res..

[25] Huang B., Zhao K., Zhang Z., Liu F., Hu H., Pan S. (2018). Changes on the Rheological Properties of Pectin-Enriched Mango Nectar by High Intensity Ultrasound. LWT.

[26] Xing Y., Xue Y., Yang X., Wang K., Li M., Wang J., Xu H. (2025). Ultrasonic Treatment Changes the Rheological Properties of Strawberry Pulp via Alterations in Particle Size and the Physicochemical Properties of Pectin. Food Hydrocoll..

[27] Arya S.S., More P.R., Das T., Hilares R.T., Pereira B., Arantes V., da Silva S.S., Santos J.C.D. (2023). Effect of Hydrodynamic Cavitation Processing on Orange Juice Physicochemical and Nutritional Properties. J. Agric. Food Res..

[28] Terán Hilares R., Dos Santos J.G., Shiguematsu N.B., Ahmed M.A., Da Silva S.S., Santos J.C. (2019). Low-Pressure Homogenization of Tomato Juice Using Hydrodynamic Cavitation Technology: Effects on Physical Properties and Stability of Bioactive Compounds. Ultrason. Sonochem..

[29] Moschakis T. (2013). Microrheology and Particle Tracking in Food Gels and Emulsions. Curr. Opin. Colloid Interface Sci..

[30] Yang N., Lv R., Jia J., Nishinari K., Fang Y. (2017). Application of Microrheology in Food Science. Annu. Rev. Food Sci. Technol..

[31] Ortega F., Ritacco H., Rubio R.G. (2010). Interfacial Microrheology: Particle Tracking and Related Techniques. Curr. Opin. Colloid Interface Sci..

[32] Bi X., Hemar Y., Balaban M.O., Liao X. (2015). The Effect of Ultrasound on Particle Size, Color, Viscosity and Polyphenol Oxidase Activity of Diluted Avocado Puree. Ultrason. Sonochem..

[33] Suo G., Zhou C., Su W., Hu X. (2022). Effects of Ultrasonic Treatment on Color, Carotenoid Content, Enzyme Activity, Rheological Properties, and Microstructure of Pumpkin Juice during Storage. Ultrason. Sonochem..

[34] Chemat F., Khan M.K. (2011). Applications of Ultrasound in Food Technology: Processing, Preservation and Extraction. Ultrason. Sonochem..

[35] Dakubu S. (1976). Cell Inactivation by Ultrasound. Biotechnol. Bioeng..

[36] Csiszar E., Szabo Z., Balogh O., Fekete E., Koczka K. (2021). The Role of the Particle Size Reduction and Morphological Changes of Solid Substrate in the Ultrasound-Aided Enzymatic Hydrolysis of Cellulose. Ultrason. Sonochemistry.

[37] Khandpur P., Gogate P.R. (2015). Understanding the Effect of Novel Approaches Based on Ultrasound on Sensory Profile of Orange Juice. Ultrason. Sonochem..

[38] Comarella C.G., Sautter C.K., Ebert L.C., Penna N.G. (2012). Polifenóis totais e avaliação sensorial de suco de uvas Isabel tratadas com ultrassom. Braz. J. Food Technol..

[39] Carneiro G.R., Pimentel T.C. (2025). Unraveling the Potential of Ultrasound Processing from the Consumer Perspective: A Review on Sensory Characteristics and Perception. Food Biosci..

[40] Wang W., Jung J., Tomasino E., Zhao Y. (2016). Optimization of Solvent and Ultrasound-Assisted Extraction for Different Anthocyanin Rich Fruit and Their Effects on Anthocyanin Compositions. LWT-Food Sci. Technol..

[41] Hu W., Gong H., Li L., Chen S., Ye X. (2019). Ultrasound Treatment on Stability of Total and Individual Anthocyanin Extraction from Blueberry Pomace: Optimization and Comparison. Molecules.

[42] Dumitraşcu L., Enachi E., Stănciuc N., Aprodu I. (2019). Optimization of Ultrasound Assisted Extraction of Phenolic Compounds from Cornelian Cherry Fruits Using Response Surface Methodology. CyTA-J. Food.

[43] Rohilla S., Mahanta C.L. (2024). Phytochemical Composition and In-Vitro Bioaccessibility of Phenolics in Different Varieties of Tamarillo (*Solanum betaceum*) Fruits: Effect of the High-Pressure Homogenization and Ultrasonication. Food Chem. Adv..

[44] Dubrovi I., Herceg Z., Jambrak A.R., Badanjak M., Dragovi V. (2011). Effect of High Intensity Ultrasound and Pasteurization on Anthocyanin Content in Strawberry Juice. Food Technol. Biotechnol..

[45] Sun J., Mei Z., Tang Y., Ding L., Jiang G., Zhang C., Sun A., Bai W. (2016). Stability, Antioxidant Capacity and Degradation Kinetics of Pelargonidin-3-Glucoside Exposed to Ultrasound Power at Low Temperature. Molecules.

[46] Neckebroeck B., Verkempinck S.H.E., Van Audenhove J., Bernaerts T., de Wilde d’Estmael H., Hendrickx M.E., Van Loey A.M. (2021). Structural and Emulsion Stabilizing Properties of Pectin Rich Extracts Obtained from Different Botanical Sources. Food Res. Int..

[47] Nutrition and Health Effects of Pectin: A Systematic Scoping Review of Human Intervention Studies. Nutrition Research Reviews. Cambridge Core. https://www.cambridge.org/core/journals/nutrition-research-reviews/article/nutrition-and-health-effects-of-pectin-a-systematic-scoping-review-of-human-intervention-studies/01BF0759F09A2BBC419F333B8B1D4FF9.

[48] Legentil A., Guichard I., Piffaut B., Haluk J.P. (1995). Characterization of Strawberry Pectin Extracted by Chemical Means. LWT-Food Sci. Technol..

[49] Wojdyło A., Jáuregui P.N.N., Carbonell-Barrachina Á.A., Oszmiański J., Golis T. (2013). Variability of Phytochemical Properties and Content of Bioactive Compounds in *Lonicera caerulea* L. var. kamtschatica berries. J. Agric. Food Chem..

[50] Kumar K., Srivastav S., Sharanagat V.S. (2020). Ultrasound Assisted Extraction (UAE) of Bioactive Compounds from Fruit and Vegetable Processing by-Products: A Review. Ultrason. Sonochem..

[51] Sharma S., Wani K.M., Mujahid S.M., Jayan L.S., Rajan S.S. (2026). Review on Pectin: Sources, Properties, Health Benefits and Its Applications in Food Industry. J. Future Foods.

[52] Saleh M., Lee Y. (2023). Instrumental Analysis or Human Evaluation to Measure the Appearance, Smell, Flavor, and Physical Properties of Food. Foods.

[53] (2013). EFSA Panel on Dietetic Products, Nutrition and Allergies (NDA). Scientific Opinion on Dietary Reference Values for Vitamin C. EFSA J..

[54] Oliveira V.S., Rodrigues S., Fernandes F.A.N. (2015). Effect of High Power Low Frequency Ultrasound Processing on the Stability of Lycopene. Ultrason. Sonochem..

[55] Alvarado J.D., Palacios Viteri N. (1989). Effect of temperature on the aerobic degradation of vitamin C in citric fruit juices. Arch. Latinoam. Nutr..

[56] Golmohamadi A., Möller G., Powers J., Nindo C. (2013). Effect of Ultrasound Frequency on Antioxidant Activity, Total Phenolic and Anthocyanin Content of Red Raspberry Puree. Ultrason. Sonochem..

[57] Valdramidis V.P., Cullen P.J., Tiwari B.K., O’Donnell C.P. (2010). Quantitative Modelling Approaches for Ascorbic Acid Degradation and Non-Enzymatic Browning of Orange Juice during Ultrasound Processing. J. Food Eng..

[58] Briggs J.L., Steffe J.F. (1997). Using Brookfield Data and the Mitschka Method to Evaluate Power Law Foods. J. Texture Stud..

[59] Giusti M.M., Wrolstad R.E. (2001). Characterization and Measurement of Anthocyanins by UV-Visible Spectroscopy. Curr. Protoc. Food Anal. Chem..

[60] Singleton V.L., Rossi J.A. (1965). Colorimetry of Total Phenolics with Phosphomolybdic-phosphotungstic Acid Reagents. Am. J. Enol. Vitic..

[61] (2012). Sensory Analysis—General Guidelines for the Selection, Training and Monitoring of Selected Assessors and Expert Sensory Assessors.

[62] (2007). Sensory Analysis—General Guidance for the Design of Test Rooms.

[63] Taylor K.A., Buchanan-Smith J.G. (1992). A Colorimetric Method for the Quantitation of Uronic Acids and a Specific Assay for Galacturonic Acid. Anal. Biochem..

[64] Mieszczakowska-Frąc M., Dickinson N.J., Konopacka D. (2025). Effect of Postharvest Ripening on the Phytochemical Composition and Antioxidant Properties of Fruits from Ten Plum (*Prunus domestica* L.) Cultivars. Agronomy.

